# A Case Report of Pulmonary Tumor Thrombotic Microangiopathy Caused by Lung Adenocarcinoma: The Importance of Clinical Diagnosis and Monitoring Disease Progression

**DOI:** 10.7759/cureus.80489

**Published:** 2025-03-12

**Authors:** Yuta Sakano, Yoko Hamakawa, Ryo Yamanaka, Atsushi Funauchi, Satoshi Marumo, Motonari Fukui

**Affiliations:** 1 Respiratory Disease Center, Medical Research Institute Kitano Hospital, Osaka, JPN

**Keywords:** chemotherapy, lung cancer, pulmonary hypertension, pulmonary tumor thrombotic microangiopathy, respiratory failure

## Abstract

Pulmonary tumor thrombotic microangiopathy (PTTM) is a rare and fatal lung complication associated with cancer. Diagnosing PTTM is challenging due to its rapid progression and nonspecific clinical or imaging findings, which often result in postmortem identification. Treatment options are limited, and long-term management remains poorly understood. This report describes a 50-year-old patient with lung adenocarcinoma who was clinically diagnosed with PTTM and showed improvement with early chemotherapy initiation. Disease activity was controlled through multiple chemotherapy regimens, and recurrence was monitored using blood coagulation status and echocardiography. This case highlights the importance of early clinical diagnosis and ongoing monitoring of disease progression in PTTM.

## Introduction

Pulmonary tumor thrombotic microangiopathy (PTTM) is a rare and fatal lung complication associated with cancer, first described by von Herbay et al. in 1990 [1]. PTTM pathogenesis involves the development of pulmonary hypertension due to the embolization of tumor cells into small pulmonary arteries. This results in intimal fibroblast proliferation within the blood vessel walls and subsequent narrowing of the vascular lumen. Poorly differentiated adenocarcinoma is the most common histological type associated with PTTM. While gastric cancer is the most frequent primary malignancy linked to PTTM, breast and lung cancers are also implicated [2]. Diagnosing PTTM is challenging because of the rapid progression and the lack of specific clinical or imaging findings, leading to most cases being identified postmortem [2]. No established treatment for PTTM exists, and even when diagnosed and treated, there are limited reports on monitoring and controlling disease progression [2,3].

A clinical diagnosis of PTTM was made in a patient with lung adenocarcinoma who presented with dyspnea, abnormal blood coagulation, and findings consistent with pulmonary hypertension. No evidence of pulmonary embolism or lymphangitic carcinomatosis was found, and chest imaging did not provide an explanation for the condition. The patient’s respiratory failure was managed with early chemotherapy initiation. Recurrence of PTTM was detected based on blood coagulation status and transthoracic echocardiography. Disease activity was successfully controlled for six months with multiple rounds of chemotherapy.

## Case presentation

A 50-year-old male patient with a history of advanced lung adenocarcinoma and multiple lung metastases presented to our hospital with worsening dyspnea. He was a former tobacco smoker with a 20-pack-year history. No driver gene mutations were detected in his cancer. His treatment history included first-line chemotherapy with cisplatin, pemetrexed, and pembrolizumab as first-line chemotherapy, followed by second-line chemotherapy with docetaxel and ramucirumab. During second-line treatment, chemotherapy was delayed for approximately one month due to the development of a perianal abscess, which required surgical intervention under local anesthesia. The patient developed dyspnea the day following surgery.

Upon admission, four days after symptom onset, the patient was alert, afebrile (36.3 °C), slightly hypotensive with reduced pulse pressure (blood pressure 106/86 mmHg), and hypoxemic (oxygen saturation 85% on room air). He exhibited tachycardia (101 beats per minute) but no adventitious heart or lung sounds. No lower extremity edema was noted. Laboratory tests revealed elevated D-dimer, C-reactive protein, brain natriuretic peptide, and carcinoembryonic antigen (CEA) levels (Table 1).

**Table 1:** Laboratory findings on admission. ALP: alkaline phosphatase, ALT: alanine aminotransferase, APTT: activated partial thromboplastin time, AST: aspartate aminotransferase, BNP: brain natriuretic peptide, BUN: blood urea nitrogen, CEA: carcinoembryonic antigen, CRP: C-reactive protein, LDH: lactate dehydrogenase, PT-INR: prothrombin time-international normalized ratio, RBC: red blood cell, WBC: white blood cell.

Complete blood count		Biochemistry	
WBC	6800/μL	Total protein	5.7 g/dL
Neutrophils	73.70%	Albumin	3.4 g/dL
Eosinophils	5.10%	AST	14 IU/L
Basophils	1%	ALT	14 IU/L
Monocytes	14%	LDH	393 IU/L
Lymphocytes	4.20%	ALP	86 IU/L
RBC	366 × 10^4^/μL	Total bilirubin	0.6 mg/dL
Hemoglobin	11.2 g/dL	BUN	19.5 mg/dL
Hematocrit	34.30%	Creatinine	1.08 mg/dL
Platelets	13.2 × 10^4^/μL	Sodium	141 mEq/L
Arterial blood gas (room air)		Potassium	3.7 mEq/L
pH	7.431	Chloride	111 mEq/L
pCO_2_	35.6 mmHg	CRP	1.96 mg/dL
pO_2_	68.8 mmHg	BNP	285 pg/mL
HCO_3_^-^	23.2 mEq/L	CEA	47.8 ng/mL
	23.2 mEq/L	Coagulation status	
		PT-INR	0.95
		APTT	30.4 sec
		D-dimer	6 μg/mL

**Figure 1:**
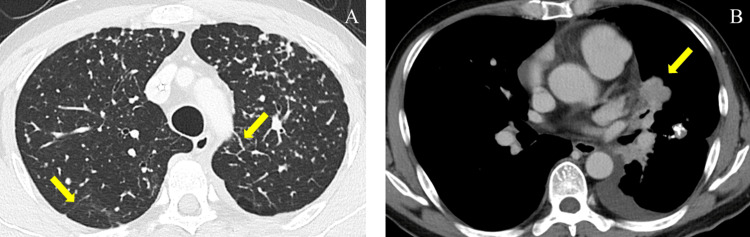
Chest computed tomography on admission. (A) Chest computed tomography imaging revealed military metastases and interlobular septal thickening in both lung fields (yellow arrows). (B) Minor pleural effusion and slight tumor progression (yellow arrow).

**Figure 2:**
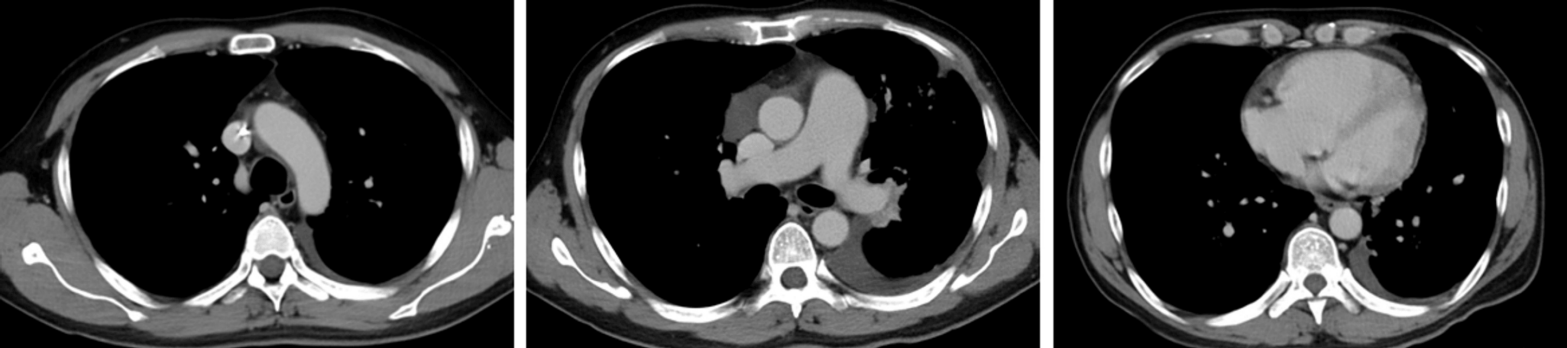
Contrast-enhanced chest computed tomography on admission. Contrast-enhanced chest computed tomography imaging showed no evidence of pulmonary embolism.

**Figure 3:**
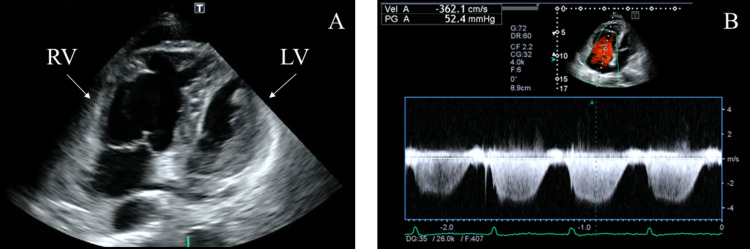
Transthoracic echocardiography on admission. (A) Transthoracic echocardiography showed right ventricular (RV) dilatation, flattening of the interventricular septum, and akinesia of the RV mid-free wall except for the apex, known as McConnell’s sign. (B) Tricuspid regurgitation with a pressure gradient (TRPG) was elevated at 52.4 mmHg.

Contrast-enhanced chest computed tomography (CT) revealed interlobular septal thickening, minor pleural effusion, and slight tumor progression (Figure 1), with no evidence of pulmonary embolism (Figure 2). Transthoracic echocardiography revealed tricuspid regurgitation with a pressure gradient (TRPG) of 52.4 mmHg, right ventricular (RV) dilatation, flattening of the interventricular septum, and McConnell’s sign, defined as akinesia of the RV mid-free wall except for the apex (Figure 3).

**Figure 4:**
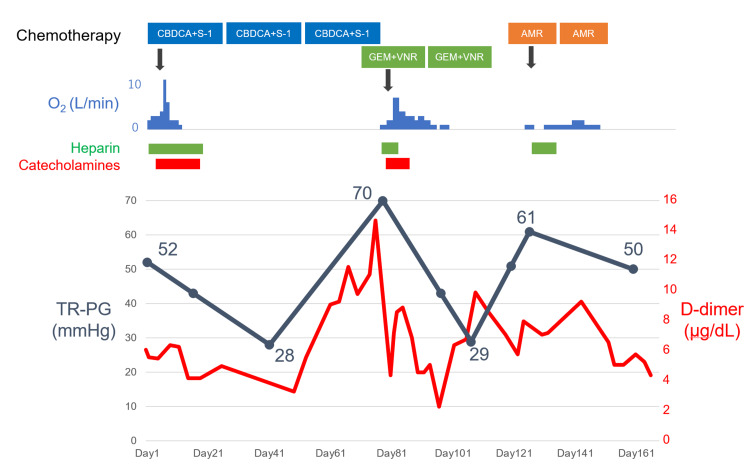
Clinical course of the patient. Clinical course of the patient during ongoing chemotherapy. D-dimer varied along with the relapse and severity of pulmonary tumor thrombotic microangiopathy. TRPG: tricuspid regurgitation with a pressure gradient, AMR: amrubicin, CBDCA: carboplatin, GEM: gemcitabine, S-1: tegafur/gimeracil/oteracil, VNR: vinorelbine.

These findings suggested acute right-sided heart failure due to pulmonary hypertension. Given the rapidly progressive pulmonary hypertension in the peripheral pulmonary arteries, without evidence of pulmonary emboli on CT, PTTM was suspected. Considering the rapid clinical progression of PTTM, third-line chemotherapy with carboplatin and tegafur/gimeracil/oteracil (S-1) was administered on the day of admission. Thereafter, the patient's dyspnea and right heart failure worsened, requiring circulatory support with dopamine, dobutamine, and oxygen therapy (11 L/min with a non-rebreathing mask). However, respiratory failure and pulmonary hypertension began to improve by the sixth day of chemotherapy. After two cycles of chemotherapy, pulmonary hypertension improved (TRPG decreased to 28 mmHg), and the patient was discharged. D-dimer levels appeared to correlate with the severity of respiratory failure (Figure 4).

**Figure 5:**
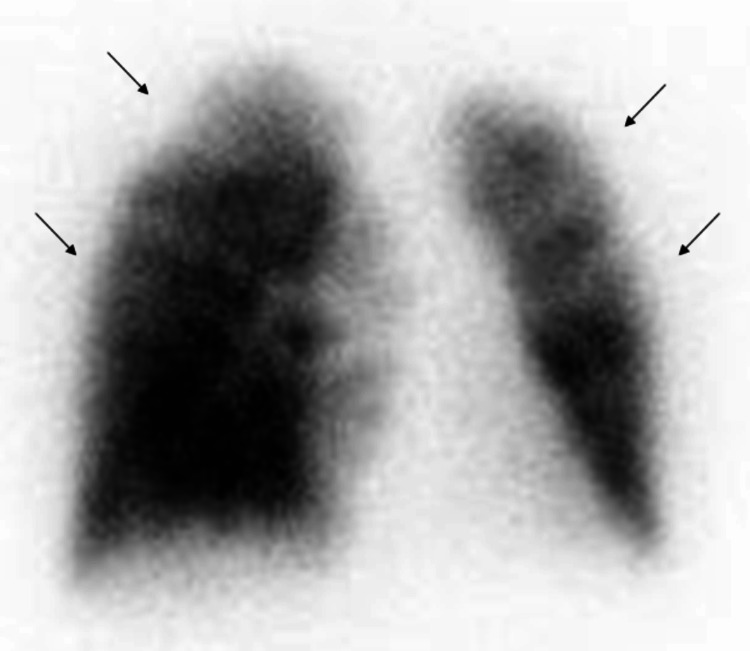
Lung perfusion scan. Bilateral multiple wedge-shaped perfusion defects in lung perfusion scintigraphy (arrows).

During three cycles of third-line chemotherapy, the patient was hospitalized for a pneumothorax, which did not result in respiratory failure, and underwent chest tube thoracostomy. Although lung expansion was successful after thoracostomy, dyspnea recurred along with elevated D-dimer levels. Transthoracic echocardiography revealed an elevated TRPG of 70.0 mmHg. A lung perfusion scan identified multiple peripheral subsegmental defects (Figure 5), indicating a relapse of PTTM.

A fourth-line chemotherapy regimen with gemcitabine and vinorelbine was initiated. Although dyspnea and right heart failure recurred, requiring circulatory support and oxygen therapy (7 L/min via a simple mask), the patient's condition improved within a few days of chemotherapy, indicating disease control. He was discharged after undergoing pleurodesis for the pneumothorax and completing chemotherapy. After two cycles, TRPG decreased to 29 mmHg, and D-dimer levels decreased in conjunction with TRPG. The size of the visible tumor remained unchanged.

Before completing three cycles of fourth-line chemotherapy, the patient developed abdominal pain due to metastatic lesions in the pancreas and abdominal lymph nodes. At the time of the visit, the patient was free of dyspnea, but TRPG had increased to 51 mmHg. Given his good performance status, fifth-line chemotherapy with amrubicin was initiated to control disease progression and the recurrence of PTTM. Respiratory failure initially worsened but improved after two cycles of fifth-line chemotherapy, allowing the patient to discontinue oxygen therapy. After two cycles of chemotherapy, TRPG improved from a peak of 61 mmHg to 50 mmHg. However, metastatic lesions in the abdomen progressed rapidly compared to the primary and metastatic lesions in both lungs, leading to obstructive cholangitis, which could not be treated with drainage due to severe duodenal edema and ascites. The patient was transferred to hospice care and died one month later.

## Discussion

In this case, we observed improvement in a patient with lung adenocarcinoma who developed progressive respiratory failure. This was achieved by clinically suspecting PTTM and initiating chemotherapy early in the disease course. Disease progression was closely monitored using blood coagulation status and echocardiography, and the patient received further chemotherapy promptly upon relapse.

Diagnostic difficulties in PTTM

The clinical challenge of diagnosing PTTM lies in its rapid progression and the lack of specific findings. PTTM is commonly associated with shortness of breath and cough [2]. Laboratory examinations show that more than half of PTTM cases exhibit disseminated intravascular coagulation (DIC), with thrombocytopenia and elevated D-dimer [4]. Chest CT imaging in PTTM often shows ground-glass opacity (GGO) or diffuse small nodules in the central lobules [4], although these findings are not specific to PTTM. While not specific, pulmonary hypertension is a relatively common finding in PTTM, with high sensitivity. It was observed in 89% (59/66) of patients who underwent transthoracic echocardiography [2]. Furthermore, differential diagnoses of PTTM include lymphangitic carcinomatosis and pulmonary embolism. Since lymphangitic carcinomatosis typically does not cause pulmonary hypertension, transthoracic echocardiography is a useful diagnostic tool for differentiation. Additionally, contrast-enhanced CT can help distinguish PTTM from pulmonary embolism. Pulmonary artery enlargement without thrombus and right heart enlargement are considered more indicative of PTTM [5].

A definitive diagnosis of PTTM requires demonstrating the presence of tumor cells in the pulmonary artery through a lung biopsy [1]. However, this procedure requires careful consideration. If the patient’s condition is stable, transbronchial lung biopsy or aspiration cytology with a right heart catheter may be an option [6]. However, patients with PTTM are often in poor condition, making biopsy procedures difficult to perform. Moreover, the median time from symptom onset to death has been reported to be three weeks [2], and the median survival time from the time the patient required oxygen support was nine days [4]. Thus, there is often no time to wait for a histopathological diagnosis. Even when PTTM is clinically suspected, a definitive diagnosis is often made postmortem (79%) [2]. Consequently, some authors suggest that biopsy is not essential for a definitive diagnosis and that the clinical diagnosis of PTTM, after excluding pulmonary hypertension due to other diseases such as pulmonary embolism, is appropriate [7,8].

Importance of early diagnosis and treatment

Early diagnosis and therapeutic intervention are important in the management of PTTM. Currently, there are no established treatment strategies for PTTM. The most common approach is chemotherapy for the primary tumor, which aims to eliminate tumor cells from the small pulmonary arteries [3]. Anticoagulation therapy and pulmonary vasodilators have also been reported as treatments, but it is difficult to prevent the disease process with these treatments alone because cancer progression is the primary cause of PTTM [2]. Some studies have reported the efficacy of bevacizumab, a vascular endothelial growth factor (VEGF) inhibitor, imatinib, and platelet-derived growth factor (PDGF) inhibitors [9,10]. However, these drugs may not be suitable for all patients, depending on the type of cancer and the location of metastases. Because chemotherapy does not have an immediate effect, it is crucial to suspect PTTM early and initiate treatment before the patient develops respiratory failure.

In this case, PTTM was suspected clinically based on pulmonary hypertension without pulmonary embolism, changes in tumor markers, or CT findings suggesting the progression of lung cancer. At the initial onset of PTTM, the patient presented with severe respiratory failure, leaving no time to wait for a definitive pathological diagnosis. Therefore, we started chemotherapy based on our clinical diagnosis. Following treatment, the patient’s hemodynamics, pulmonary hypertension, tumor markers, and DIC-related parameters improved, retrospectively confirming the accuracy of the PTTM diagnosis.

Assessment of disease progression in PTTM

Another clinical challenge in managing PTTM is the limited information available on long-term observation and monitoring of disease progression [2]. In this case, the patient experienced recurrent PTTM as the tumor progressed. Increases in D-dimer levels preceded the development of respiratory failure and pulmonary hypertension, and these levels decreased with chemotherapy. Given that the primary mechanism of PTTM involves vascular endothelial damage and activation of the coagulation system [4], it is reasonable to expect that D-dimer levels would rise as the embolism progresses. We monitored PTTM recurrence based on coagulation markers and pulmonary hypertension, as estimated by echocardiography. Although the patient finally died, he survived for approximately six months from the first onset of PTTM and was able to spend two of those months at home with his family. If the patient had not developed a pneumothorax, he would have stayed home for a longer time.

## Conclusions

PTTM is a fatal lung complication associated with cancer, which progresses rapidly and is difficult to diagnose. Therefore, when cancer patients present with rapidly progressive respiratory failure and pulmonary hypertension, examinations such as blood tests for tumor markers and D-dimer, echocardiography, and contrast-enhanced CT should be performed to consider the possibility of PTTM. If respiratory failure progresses rapidly, early therapeutic intervention with anticancer treatment may improve the patient’s condition. This case demonstrates the potential for monitoring and controlling PTTM progression using D-dimer levels and echocardiography.
